# Agent-based modeling: a systematic assessment of use cases and requirements for enhancing pharmaceutical research and development productivity

**DOI:** 10.1002/wsbm.1222

**Published:** 2013-06-04

**Authors:** C Anthony Hunt, Ryan C Kennedy, Sean H J Kim, Glen E P Ropella

**Affiliations:** 1 Department of Bioengineering and Therapeutic Sciences, University of CaliforniaSan Francisco, CA, USA; 2 Department of Pharmacy and Therapeutics, University of PittsburghPittsburgh, PA, USA; 3 Tempus Dictum, Inc.Portland, OR, USA

## Abstract

A crisis continues to brew within the pharmaceutical research and development (R&D) enterprise: productivity continues declining as costs rise, despite ongoing, often dramatic scientific and technical advances. To reverse this trend, we offer various suggestions for both the expansion and broader adoption of modeling and simulation (M&S) methods. We suggest strategies and scenarios intended to enable new M&S use cases that directly engage R&D knowledge generation and build actionable mechanistic insight, thereby opening the door to enhanced productivity. What M&S requirements must be satisfied to access and open the door, and begin reversing the productivity decline? Can current methods and tools fulfill the requirements, or are new methods necessary? We draw on the relevant, recent literature to provide and explore answers. In so doing, we identify essential, key roles for agent-based and other methods. We assemble a list of requirements necessary for M&S to meet the diverse needs distilled from a collection of research, review, and opinion articles. We argue that to realize its full potential, M&S should be actualized within a larger information technology framework—a dynamic knowledge repository—wherein models of various types execute, evolve, and increase in accuracy over time. We offer some details of the issues that must be addressed for such a repository to accrue the capabilities needed to reverse the productivity decline. © 2013 Wiley Periodicals, Inc.

## INTRODUCTION

Pharmaceutical research and development (R&D) is in the midst of a productivity decline. Unified, transdisciplinary, in silico modeling and simulation (M&S) methods are viewed broadly as a promising countermeasure. Agent-based (AB) modeling is a phrase used currently to identify relatively young modeling methods that utilize software agents. We review and present evidence that AB methods will be essential contributors to successful M&S countermeasures.

## TRI-FOCUS: AGENT-BASED M&S, R&D, AND EXPLANATORY MECHANISMS

The development of agent modeling tools and the availability of increasingly detailed, varied, and abundant data coupled with advances in computation have made possible a growing number of agent-based modeling and simulation (AB M&S) applications across a variety of non-biomedical domains and disciplines. To illustrate, we identify early^1^–^15^ and more recent^16^–^32^ use cases. Macal and North^33^ provide additional examples and a tutorial on AB M&S methods. Within the biomedical domain, AB M&S is a relatively new approach for studying systems composed of interacting components, some of which can be autonomous. The methods are used primarily to gain insight into mechanisms responsible for phenomena of living systems. We cite early^34^–^40^ and more recent^41^–^56^ examples of such applications. Amigoni and Schiaffonati^57^, An et al.^58^, and Edelman et al.^59^ provide biomedically-focused reviews. Several applications exemplify the expanding variety of applications relevant to the pharmaceutical sciences.^46^,^60^–^72^

Pharmaceutical science stakeholders agree that there is a crisis within the broad pharmaceutical R&D domain (private and public): productivity continues to decline, even in the face of dramatic scientific and technical advances accompanied by a data deluge, especially at the molecular level. A flurry of recent reviews and commentaries^73^–^102^ discuss the problems from several different perspectives and offer strategies and scenarios for how M&S methods can and are being used to enhance productivity. The different perspectives include pharmacometrics [including translational and physiologically based pharmacokinetics–pharmacodynamics (PBPK), and disease progression M&S], quantitative and systems pharmacology, and model-based drug development. Given the apparent fit between designed uses of AB M&S methods and the multifarious problems cited in the reviews that can benefit from computational M&S methods, it is surprising that only two of the citations mention AB M&S methods. No examples are presented within those citations of any current use of AB M&S methods within pharmaceutical companies. Does that situation signal ripe opportunity for AB M&S methods to add new value beyond that delivered using the established methods? That question, although provocative, is premature because one should not try to force-fit a particular M&S method to a particular set of problems. We should first evaluate where and how the cited domain experts envision M&S methods being used to reverse the productivity trend. We can then select those M&S methods that enable those uses.

The cited experts anticipate shifting focus from analysis of data to discovery and challenge of explanatory mechanisms. They see M&S becoming integral and essential to envisioned discovery and development processes that are dramatically more productive in part because of improvements in use of M&S methods across all R&D activities. Yet they also make clear that the established practice is to select one or more modeling tools, drawn primarily from those already in use,^75^ to address the particular problem at hand. The practice is the same independent of stage in the R&D process. Various scientists, often separated in time and space across R&D processes, integrate the derived information. Anticipating that having M&S integrated across R&D activities can improve productivity, what new demands are placed on M&S methods? We show why multifaceted, networked M&S use cases require drawing from an expanded computational modeling method and tool repertoire. Can current methods and tools fulfill the requirements? No. We justify that answer and discuss why AB and more advanced M&S methods are essential. It becomes clear that an analog (see Box 1) based knowledge repository will be needed. We identify M&S method requirements, and that brings into focus additional M&S use cases that we believe the repository's framework will likely need to enable.

**BOX 1**

**DEFINITIONS**

*Actor*: an entity identifiable by an observer as a cause of an effect; an entity that participates in a process (plays a part); in computer science it is a mathematical model of computation that treats *actors* as the universal primitives: in response to a received message, an actor can make local decisions, create more actors, send messages, and determine how to respond to the next message received.

*Agent*: a software object that can schedule its own events (within an analog, it is quasi-autonomous); it senses and is part of its environment; it pursues and can revise an agenda within a larger script; some of its attributes and actions may be designed to represent biological counterparts, whereas others will deal with issues of software execution.

*Agent-based*: something formulated with or built up from agents; in this context, it identifies a simulation model in which quasi-autonomous agents are key components. Terms that are often synonymous within the M&S literature include individual-based and multi-agent.

*Framework*: a carefully crafted assemblage of tools, devices (some software, some hardware, and occasionally even some wet-ware), usage protocols, good practices, etc., governed by a set of component interoperability standards. Upon satisfying use cases and listed requirements, we expect the framework to be an extensible, distributed, open, and loosely coupled (yet unified from the user's perspective).

*Analog*: a software device that has (some) aspects and attributes that are similar to those of its R&D referent yet can exist and operate in isolation and in the absence of its R&D counterpart; in biomedical M&S, a model implemented in software that, when executed, produces phenomena that mimic one or more attributes measured or observed during referent wet-lab experiments. In this context, most analogs will be suitable for experimentation.

*In silico experiment*: it is precisely analogous to a wet-lab experiment. An analog is a hypothesis: the mechanism produced upon execution by interacting components will result in phenomena, often at different scales, that are similar, or not, to prespecified wet-lab phenomena. Measurement of features during execution enables testing the hypothesis. That activity is an *in silico* experiment.

*Analog based knowledge repository*: easily accessible, organized framework feature. Its content is an up-to-date instantiation of all relevant mechanistic knowledge in an accessible, easily understood, observable, and interactive form. It contains annotated records of analogs (current and falsified), their mechanisms, how they were composed plus the rationale, along with records of *in silico* experiments. All use cases (see ‘Paramount use cases’ subsection) can be achieved by employing the analog based knowledge repository. We envision domain experts making go/no-go decisions after interactive exploration of many scenarios (simulations) within the repository.

## MODELING, SIMULATION, AND THE PRODUCTIVITY CRISIS

Ideally, one should begin any M&S project without bias for any particular model types or methods.^103^ Selecting M&S methods or tools in advance of specifying uses cases can constrain and even bias thinking about explanatory mechanisms. Hence, the first task is to clearly identify near and longer term expectations. Among the questions to be answered are these: what are the problems? What questions need to be answered? What new knowledge is sought and how will it be used? What decisions must be made? When are the deadlines? What resources are available? etc. Within pharmaceutical R&D, these questions are complicated by the nested, networked, multi-year, evolving nature of the overall enterprise. Scientific insight achieved at an early stage may be critical downstream (e.g., during dose ranging studies or clinical trials), and early planning for that prospect may influence selection of M&S methods in important ways. Yao et al.^102^ illustrate the variety of relationships and common computational methods that support them (see Figure S1 in Supporting Information).

Consider a model and method judged appropriate for a particular discovery or early development problem. The insight sought is intended to be useful for downstream as well as for current decision-making. Can the model, method, and information be easily utilized as needed within a tight time window at a downstream go/no-go decision juncture? If doing so proves problematic because of differences in models, methods, and/or tools, or perhaps because of inadequate records of analogs and (more notably) their *in silico* experiments, etc., uncertainties and delays increase unnecessarily along with the risk of a flawed decision. The situation is complicated further by a larger, pressing, overarching need: reverse the declining trend in productivity.

**FIGURE 1:**
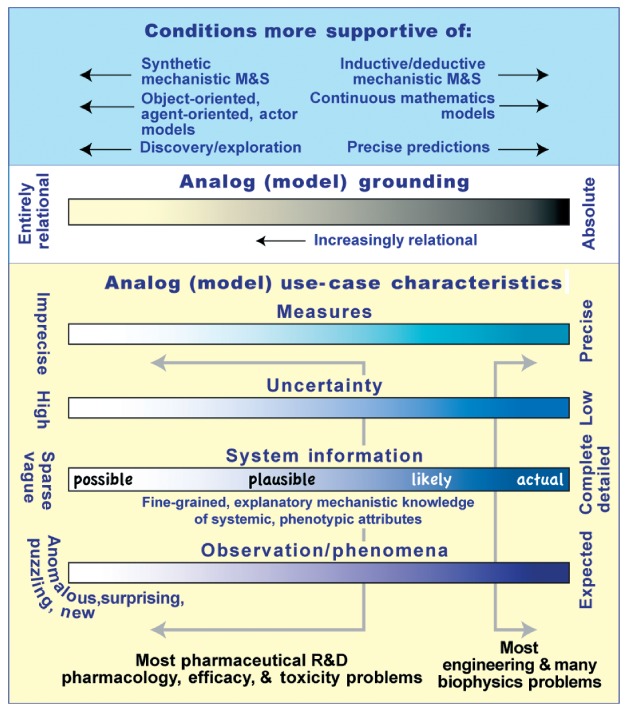
A particular analog use case can be characterized by an approximate location on each of the four lower spectra. Spectrum location along with specifics about how and for what the simulation model will be used within the larger R&D context, determine which M&S methods are most appropriate for a given use case. Within a pharmaceutical R&D context, a use case includes details of the specific, biology-focused wet-lab experiment that analog execution is intended to model in some way. Depending on use case location on the spectra, an analog can be located anywhere along a spectrum of software devices (models) ranging from synthetic (all components designed to be plugged together and are thus replaceable) to purely inductive models. *System* *Information* includes current conceptual knowledge about the mechanisms on which wet-lab experiments focused. As the R&D process advances, evidence will shrink the space of possible mechanisms. The result will be a set of plausible analog mechanisms supported by validation evidence. Later, that set too will shrink. The result will be a smaller set of likely mechanisms (those that have survived several falsification experiments). An R&D project's product can be successful without being able to designate key mechanisms as either actual or likely. Actual mechanisms are typically known for engineered systems, but are typically lacking in therapeutics. Grounding is discussed in Box 2; additional information is provided as Supporting Information. Conditions on the far right of the bottom four spectra are supportive of models (typically, continuous equations) that rely exclusively on absolute grounding. Hunt at al.^104^ make the case that when left of center on one or more of the bottom three spectra, models should rely more on relational grounding. When on the far left, early stage, purely qualitative M&S is still useful and productive: it facilitates goal-oriented research efforts by clarifying (unifying) current thinking about referent phenomena. Such models would typically be coarse grain and use relational grounding. Spectra colors were selected arbitrarily.

The cited experts make the case that M&S methods are essential to simultaneously increasing productivity of therapeutic drug development, and facilitating the recursive cycle of new knowledge buildup aimed at improved quality care. Accepting that position, we view the enterprise as a networked, experiment-intensive system that begins small (significantly left on the Systems Information spectrum in Figure 1). As R&D progresses, the system evolves and expands. It becomes a large, distributed, multiscale, multi-aspect M&S challenge. For a particular aspect of the system, its location on the Systems Information spectrum in Figure 1 inches to the right. In what variety of ways do the cited experts envision M&S methods being used? Answers are provided in Supporting Information-Use Cases; a sample of answers is provided in Box 3. We propose a set of seven paramount use cases that are derived from and subsume those particular use cases. We then present five requirements, which we argue must be satisfied to enable achieving those paramount use cases efficiently. We discuss M&S methods that will enable a unified M&S approach to satisfy those requirements. To our knowledge, such an analysis has not been done. We argue that such an examination is necessary and essential to discover strategies capable of restoring and enhancing productivity.

## INTEGRATIVE MANAGEMENT OF KNOWLEDGE AND UNCERTAINTY

Knowledge integration is an essential, paramount use case. For this discussion, we limit attention to knowledge relevant to the primary R&D project focus: the particular disease, morbidity, or health issue; the biologic or chemical entity treatment intervention; the pharmacological, toxicological, and clinical outcomes that are consequences of treatments; and the variety of mechanisms (even when vague) that are offered to explain those phenomena. We ignore other important categories of knowledge that can impact go/no-go decisions, but the approach and framework described below can be expanded to include them. Examples of those categories include regulatory science; chemistry, manufacturing, and control; human resources; accessible contract services; etc.

The cited reviews discuss roles of various computational models and methods in knowledge integration. However, we explain below that little knowledge actually resides in those models. Much of the mechanistic insight resides within mental models, and that presents a problem: mental model differences, similarities, and inconsistencies are difficult, and often impossible to ascertain. Mental models are subject to their own forms of error introduction and propagation. Increasing reliance on synthetic analogs (defined below under Mechanism-Based Approach) presents advantages because they can evolve into executable representations of what we know (or think we know) about biological systems. Those representations are called executable biological knowledge embodiments^64^,^65^,^104^ and dynamic knowledge representations.^34^,^58^,^60^,^61^,^105^–^109^ Such executions are suitable for knowledge discovery.^58^,^108^,^109^ Knowledge embodiment is made feasible because synthetic analogs provide concrete instances of that knowledge rather than analytic descriptions of conceptual representations. When an analog is executed, it demonstrates when, how, and where our knowledge matches or fails to match referent system details, which enables and facilitates knowledge discovery.^58^,^108^,^109^

For current knowledge and beliefs to be useful (especially in a social context like shared model usage, validation, and falsification), it must be embedded in an analog and visible to the user (which is often not the case now). Analogs help build schemata for knowledge (and ignorance) representation, which can provide a mechanism for the curation and maintenance of the embedded knowledge. Users must be able to readily identify the knowledge and be able to discuss it, rely on it, dispute it, and falsify it (or not), all while reflecting on how that knowledge impacts the project. To achieve these capabilities, most, if not all, modeling activities and all mechanism representations will need to take place within a framework of the type described in Box 1 and discussed below. The R&D project related content of that framework will become part of an interactive, analog based knowledge repository.

It should be noted that knowledge is not embedded in the any of the variety of pharmacometric or systems pharmacology models identified in the reviews.^73^–^102^ Model refers specifically to the equations used to describe the referent aspects, dynamics, and features^75^,^79^,^84^–^90^,^93^,^95^ as implemented in software. As documented by An^105^,^107^ and Hunt et al.^64^,^104^ embedding knowledge in pharmacometric and ordinary differential equation (ODE) models is challenging, if not problematic. Equations are typically used to describe patterns in data. The associated conceptual models do reflect knowledge, but it exists separate from the equations. Humans interpreting the I/O of the cited equations use prose and sketches to provide the semantic grounding for those models in terms of both the idealized, conceptual model and knowledge of the referent system. That semantic grounding is done manually, and is separate from the equations and their software implementations. Hence, neither knowledge nor semantics is *embedded* in those equations. However, the equation models are typically encapsulated within ‘ready to use’ software tools and packages. Bouzom et al.^75^75 lists and describes the more frequently used general and constrained tools and packages. The builders, especially of the constrained, domain-focused tools and packages, invest considerable effort in collecting, organizing, and enabling use of domain knowledge, information, and data related to a variety of model use cases. So doing considerably lowers the barrier to model parameterization and scenario exploration. The preceding points are expanded upon in Supporting Information using two concrete examples (Box 2).

**BOX 2**

**ANALOG GROUNDING ISSUES**

Grounding issues must be addressed to satisfy the listed requirements. Key issues discussed in detail by Hunt et al.^104^ are summarized in Supporting Information. The units, dimensions, and/or objects to which a variable or model constituent refers establish groundings. Absolute grounding: variables, parameters, and input–output (I/O) are in real-world units. Relational grounding: variables, parameters, and I/O are in units defined by other system components. Relational grounding requires a separate analog-to-referent mapping model. To satisfy requirements, a spectrum of model classes, methods, and groundings (illustrated in Figure 1) will be needed: absolute grounding occupies one extreme; relational grounding occupies the other.

Absolute grounding provides simple, interpretive mappings between simulation output, parameter values, and referent data. However, complex issues must be addressed each time one of the following occurs: expand the model to include additional phenomena; combining models; and/or model context changes. Expansions are challenging, even infeasible, when center-left in Figure 1. Reusability is hindered in part because the conflated semi-mechanistic, equation-based model and the model-to-referent mapping model have different use cases.

Analogs must evolve (become more complicated) as R&D advances and new mechanistic insight accumulate. That evolution will require changing, adding, and removing component linkages within analogs. We recommend keeping most component groundings relational. So doing facilitates component replacement, limiting any one component formulation solely to its coupling with the others. Any component can be replaced at will as long as the minimal I/O interface requirements are met. Starting with relationally grounded analog components allows the modeler to iterate progressively from qualitative to quantitative validation.

### Acknowledging and Reducing Uncertainty

We focus on uncertainties directly associated with the mechanistic theories on which the project is based. At the start of a typical project, uncertainty is pervasive. Consequently, projects typically begin considerably left in Figure 1 Uncertainty and System Information spectra. Uncertainty sources are varied and plentiful. They need to be identified, annotated, and updated. As the project advances successfully, we inch to the right on both the System Information and Uncertainty spectra. Productivity-enhancing M&S methods must facilitate and increase the pace of that movement in real time. We can infer that new M&S methods will be needed that are capable of generating new knowledge without requiring new wet-lab experiments. We can also infer that they must also be capable of explorartion and advanced selection of strategies to reduce uncertainties systematically in real time in parallel with advancing wet-lab experiments. Preference must be given to M&S methods that can do both.

Reliance on computational models that simply abstract away uncertainties is counterproductive: so doing does yield simple, easily managed models, which can be useful for particular use cases, but it also adds a new source of uncertainty. The core source of uncertainty is the experimental evidence on which the project's mechanistic theories are based (in the context of all other available knowledge and insight), and we recognize that current theory at a particular project stage is just one drawn from a space of possible or plausible mechanistic theories.

Studies conducted by Amgen^110^ and Bayer HealthCare^111^ found that published, preclinical, scientific findings that were important to their R&D efforts could be confirmed in only 11–25% of cases. A rule-of-thumb among early-stage venture capital firms is that at least 50% of published studies, even those in top-tier academic journals, cannot be repeated.^112^ Consequently, there is significant risk that the evidence supporting the conceptual mechanistic models on which the project is based is flawed (even if the theory proves reasonably correct). Even when experiments are repeated, variability of results can be considerable. Rarely can measurements made on cell culture models be mapped quantitatively 1:1 to comparable measures made on animal models. Similarly, animal model phenomena rarely map 1:1 to human counterparts. Hence, there is considerable uncertainty about how results from different experiments can or should be mapped to—and thus influence—the conceptual mechanistic models on which project scientists are relying. Such uncertainty and mapping dilemmas present problems for those using conventional inductive, equation-based models (typically ODEs) of the type discussed in the cited reviews. Modelers understand the issues: when using such methods, they would prefer to be on the far right of Figure 1. The conventional strategy is to request more data. So doing postpones mechanistic modeling. Unfortunately, it may be necessary for the project to advance beyond the next few go/no-go decision points before the requested data begins to come available. While waiting for the required data, the accepted strategy for dealing with uncertainty issues is to abstract them away: if we make particular simplifying assumptions, we can create an idealized, hypothetical mechanistic scenario that, if fully validated, will place us on the right side of Figure 1 where inductive, predictive models can be reliable. In doing so, model-grounding issues, discussed below, are typically ignored. As the project advances, there is no straightforward way to ‘add back’ the various uncertainties abstracted away, even if they could be measured, or undo the simplifying assumptions.

Analogs are particularly useful in managing uncertainty because they can be used to simulate possible and plausible mechanisms when center-left on the three lower Figure 1 spectra (although our focus here is on uncertainty, other elements that are often also abstracted away, such as biological or experimental details, may also be handled more concretely by analogs). An analog's mechanism is a consequence of components interacting during execution. Monte Carlo variations in component specifications, rules specifying interactions, and parameterizations can cause phenomena generated during each execution to be unique. That process simulates the non-deterministic nature of biological phenomena. That variety can also represent uncertainty about generative mechanisms, experimental variability, and intra- and interindividual variability. The challenge then becomes to follow a disciplined protocol, as done in Sheikh-Bahaei and Hunt^71^71, Hunt et al.^109^, and Lam and Hunt^113^, to identify and apply constraints in conjunction with *in silico* experimentation (see Box 1) to systematically shrink the space of acceptable Monte Carlo variations. Having multiple, equally satisfactory analogs of the same referent is an acknowledgment and representation of uncertainties that can shrink but not vanish.

### Avoiding Information Loss, ‘Warts and All’, is Essential

A product of modeling efforts cited in the reviews, irrespective of model type, is derived measures that are recorded for use by others. Examples include mean predicted ED50 (±SD), clearance, half-life, volume of distribution, etc. Examples described in Supporting Information-Use Cases^73^–^102^ illustrate that derived measures acquired during an early R&D stage can influence the perspective taken or focal aspect of interest at a later stage. However, derived measures are lossy (information is lost). Features present in the raw experimental observations are lost. Contextual information, including experiment method details, assay information, etc., may also be lost. Information loss is a concern: later, it may prove critical. Similar to issues discussed above, it becomes increasingly infeasible to recover lost information. Today, preventing such information loss can be challenging: proper measures are required to preserve, manage, and transfer information. During a project's lifetime (and thereafter), it requires attention to those preservation details across different functional groups through each development stage, from preclinical to postmarketing. The risk of critical information loss increases for conceptual model descriptions enriched with quantitative, mechanistic, pharmacological, physiological, and systems biology details that do not readily reduce to simple, mathematical or statistical descriptions.

Despite the above concerns, we stress that derived measures along with highly abstracted models are essential: researchers need them. They provide a much needed bridge from particular and specific concrete modeling to the more powerful generalized models from which researchers will develop the theory for therapy controlled normal-to-morbid or diseased-to-normal transitions. For this reason, the framework must facilitate access to, and the analog based knowledge repository must house, curate, and facilitate access to analogs, metadata, and much of the experimental observations from which the metadata were derived.

Consider measures derived from multiple different models separated in time, combined and extended beyond their original model use cases, and how they may influence downstream conceptual models used for go/no-go decision-making. Conceptual models can be expected to push the go/no-go decision in one direction. A quite different direction may result if all the original models, including those that at an earlier time were judged ‘failures’, could be re-run together to directly inform decision makers when that decision is required. An analog based knowledge repository fulfilling the requirements presented below will guard against information loss and knowledge distortion. Having intuitive, easily understood analogs will reduce dependency on, and usefulness of, lossy, derived measures to inform domain experts' conceptual models.

## EFFECTIVE DECISION-MAKING SUPPORT REQUIRES A UNIFIED FRAMEWORK

Today, domain experts rely primarily on their own conceptual models—informed, of course, by computational models—to make go/no-go decisions. Improved productivity can be achieved by changing that relationship: domain experts make go/no-go decisions after using simulations to explore many scenarios within a computational framework built and equipped specifically for scenario exploration in addition to enabling the multiple M&S methods needed to satisfy the requirements below. It will be the ‘framework’ within which all model use cases occur. We envision the framework becoming an **analog based knowledge repository**: an up-to-date instantiation of all accumulated, new, and proprietary mechanistic knowledge in an accessible, easily understood, observable, and interactive form. As a consequence, R&D project team members will have reduced dependency on the difficult to challenge conceptual mechanistic models of current, past, and no longer available domain experts.

Current experiment records and/or protocols capture and preserve some current, underlying, mechanistic conceptualizations, however, that contextual information is typically decoupled from data and can become unavailable in subsequent phases of development. A good practice will be that information is documented and provided as part of annotation within the framework. It will not be ancillary, as in OpenABM.^115^ In order to effectively incorporate data, especially wet-lab data across discovery and development stages for future use, automated capabilities will be needed that enable and facilitate metadata annotations, which may include biological, anatomical, and physiological details across biological scales. Of course, the framework will also provide methods for storage, curation, composition, and execution of analogs representing what is known about domain-specific referent systems. Its core constituents (specific items) will be data, semantic relationships, workflow actions, and computational components. There will also be a variety of derived constituents including data sets, semantic networks (e.g., XML ontologies), workflows, plus analogs and their components. Under Resources, we list currently available tools that can be used to organize and complete all of the preceding tasks.

As an example of a knowledge repository decision support use case, consider a task to estimate the likelihood of success for a clinical trial given a compound and cohort sample specifications. The following may seem futuristic, yet tools for enabling all capabilities (listed under Web Resources) are in use today within different domains. The project's inter-disciplinary team, including regulatory scientists, statisticians, programmers, regulatory staff, etc., populate the knowledge repository with experimental protocols (Workflows under Resources), experimental data sets, analog mechanisms (Executable models under Resources) representing the cohort and compounds, and maps between the terms (objects, methods, variables, parameters, graphs, etc.) used in all these elements. Software agents and actors within the repository simplify the process. A user specifies a set of objectives or criteria for a successful (or unacceptable) clinical trial outcome and assembles at least one analog that might plausibly generate data satisfying the objectives. Repository agents facilitate the composition by periodically checking the consistency and completeness of the evolving analog. The user then executes the analog according to study design workflow(s). Execution results will exhibit the systemic causation and variability generated by fine-grain, networked events embedded in analog components. Repository agents will compare and contrast the results to the objectives and present the user with similarity scores validating or falsifying analogs. Repository agents may also present a suite of alternatively composed analogs consistent with the data, ontologies, and workflows previously installed. Similarity scores provide a rudimentary estimate of the likelihood of success for the clinical trial. Further execution and similarity scores of alternative analogs provide refinement of and confidence (or the lack thereof) in those estimates. Note that because we are left of center in Figure 1, the approach avoids assuming the existence of a perfectly accurate analog. Any analog that achieves all face validation targets and satisfies the various similarity measures will be considered valid (until falsified). We envision all of the preceding being completed within hours.

As a technical note, an analog may contain components based on different formalisms. Some may be graph theoretic. Some may use ODEs. Others will be (or will use) agents. The preceding discussions illustrate that referring to the modeling activities as being AB is misleading. Agent-directed or agent-oriented are more accurate descriptions.^114^

## FROM PARTICULAR TO PARAMOUNT M&S USE CASES

We worked through each of the cited reviews and commentaries^73^–^102^ and identified more than ninety general and specific M&S use cases. Although each article presented its assessment from a particular perspective, there was, as might be expected, considerable overlap in specific use cases. That overlap guided and facilitated clustering them into seven categories that span all pharmaceutical R&D activities. Given use cases, we sought general requirement statements that would subsume two or more uses within each category. They are listed in the subsequent section.

### Paramount Use Cases

The following are called paramount M&S use cases because they subsume the particular use cases in Supporting Information-Use Cases clustered under the following seven categories.

#### Knowledge Integration

Given multiple organizational perspectives (disease state, prognostic factors, drug characteristics, cohort variability, disease progression, drug effects, timelines, budgets, etc.) and multiple data sets (qualitative and quantitative; some possibly incommensurate) from various experiments and experimental models, solve for a collection of alternative, composable, explanatory mechanisms parameterized by, and validated against, available data. Here, ‘solve’ is defined as applying constraints within an iterative protocol (see e.g., Tang and Hunt^116^, Park et al.^117^, Engelberg et al.^118^) to shrink a set of possible mechanisms into a much smaller set of plausible (supported early by qualitative validations) and incrementally more likely mechanisms (supported by quantitative validation).

Use collected mechanisms to assemble concrete, consistent, comprehensive, clinically relevant ‘stories’ about the co-evolution, during and following treatment, of subject (multiscale), the condition being treated, and treatment as well as (absorption, distribution, metabolism, and elimination) ADME plus response monitoring in the case of a drug. In this context, a story is the narrative created during simulation, and a simulation results from executing an *in silico* experiment. There are several reasons why a good simulation story is important.^119^,^120^ Use assembled stories to discover and make explicit potential conflicts, voids, and ambiguities within an analog based knowledge repository, thereby providing immediate predictive and analytical use, while bringing into focus paths for repository improvement.

#### Management of Uncertainty

Present for *in silico* experimentation, plausible mechanisms exhibiting variability, both composite and singular, that are qualitatively and quantitatively similar to that seen (or expected) in laboratory experiments or clinical trials, including matching changes in variability across different cohorts and study designs. Use simulations to estimate probability of achieving clinical target efficacy outcomes, and when feasible, estimate probability of specified, undesired effects.

#### Decision Support

Present contextualized assemblies of plausible mechanisms parameterized to provide an evidence-based justification for componentized estimates of likelihood of success at critical stages within the development process. Use the assemblies and their stories to estimate efficacy and safety windows, including confidence intervals, and to demonstrate sample sizes within appropriate population cohorts needed for constraining study outcomes to within those windows.

#### Preclinical and Clinical Development

Preclinical and clinical development along with postmarketing uses are covered by requirements listed above and below.

#### Drug-disease Modeling

Solve for plausible mechanism composites satisfying multiple long-term aspects of diseases, drug effects and fates within cohorts, placebo effect, and disease modifying interventions. Use those mechanisms to predict compound behavior (PK) within contexts of interest, including hypothetical mechanisms for lack of adherence, dropout, and multiple drug interaction.

#### Drug–drug Interaction, Special Populations

Solve for plausible mechanism composites of compound interactions of interest, including time-dependent inhibition, induction, and competition between a parent compound and its metabolites. Solve for plausible mechanism composites for various cohorts (animals, children, and adults) based on classifications of compound behavior in each cohort. Hypothesize and build mechanism translation maps between analog cohorts (e.g., between adults and children). Use plausible translation maps to design clinical trials for human cohorts.

#### Prediction

Solve for plausible mechanism composites by multi-objective search within constraints defined by fine-grained classifications of cohorts (particular attributes at multiple levels and scales) and compounds (e.g., particular physicochemical properties), including their formulation, across model types (*in vitro* to *in vivo*, animal to human). Use the plausible solutions to predict the outcomes of precise and particular regimens on individually characterized cohorts, both across (translation) and within model types.

## REQUIREMENTS

To enable all paramount use cases, the framework must enable, and analog systems must meet, these five requirements.

1. An analog's components and spaces will be concrete (enabling knowledge embodiment), wherein its details will be directly defined by its use cases. Analog components will be somewhat modular, in schedule as well as state. So doing helps accomplish the following framework activities.
  - It enables defining and annotating component- and module-to-biological counterpart mappings, making them explicit, intuitive, and easily understood. It enables experimentation on concrete analogs. It is essential for wet-lab R&D scientists and decision makers to easily follow, interpret, and comment, unassisted, on simulation details. For that, the embodied knowledge needs to be easily accessible, which requires transparency in representation and execution—form and function.
  - It enables making modules quasi-autonomous and thus more biomimetic.^65^,^104^ Analog components composing virtual cells, organs, animal models, and ultimately individuals, must exhibit some level of autonomous behavior in order to improve similarity with biological counterpart phenotypes, while increasing their explicative and predictive utility.
  - Components can be adapted easily to represent different past and future experiment designs and protocols.
  - It is straightforward to change mechanistic detail (granularity, resolution) to simulate additional attributes or experiments. It facilitates scaling (translation, morphing) among *in silico* experimental systems to represent transitions from *in vitro* to animal models, from animal model to human cohorts, and from normal health to morbid.
  - It enables reusing analogs and components along with their embedded knowledge, for study of new chemical entities (or biologics) and new intervention scenarios under similar or different morbidity constraints or epigenetic influences.
  - It facilitates verification through unit testing, where each component can be tested in isolation as well as in the composed analog context.
  - It facilitates versioning, where each component can evolve independent of other components.
  - It enables building trust in surviving analogs by accumulating direct *in silico*-to-wet-lab validation evidence, where measures taken during *in* *silico* experiments are mapped quantitatively to counterpart measures taken during wet-lab experiments. So doing reduces reliance on derived measures, which can mask information loss, assumptions, and uncertainty removal. Many of those validation exercises will rely extensively on pharmacometrics and conventional modeling methods.
  - It facilitates archiving analog and mechanism evolution along with *in* *silico* experiment successes and failures within the framework. The latter is particularly important. When an analog or *in silico* experiment fails in some way, we acquire new knowledge, e.g., a feature of an analog mechanism thought to have a particular *in vitro* biological counterpart, does not. However, in a different context, that mechanism or some variant may prove useful.
2. Components and spaces can be assembled easily to simulate current, past, and future laboratory or clinical experiments. Generating many alternative, plausible, testable (through *in silico* experimentation) components for each function/structure and then selecting against those that fail, is needed to insure generation of new knowledge. So doing helps accomplish two framework activities.
  - It becomes increasingly easy to construct (plug together) and explore alternative mechanistic hypotheses and intervention scenarios. It facilitates contrasting their predictions during simulation. So doing can help avoid dictating ‘best methods’ prematurely. The latter keeps the door open for yet-to-be-stated M&S use cases while increasing opportunities for serendipitous insight and/or discovery.
  - It becomes increasingly easy to construct multiscale, multiresolution, multi-attribute analogs (eventually individualized virtual patients) composed of heterogeneous (form, function, methods, formalisms, etc.) components.
3. Simulation experiments are feasible in the presence and absence of chemical entity objects (hereafter, CE-objects). They are also feasible in the presence of multiple CE-objects. Components within analogs can recognize different CE-objects and adjust their response accordingly.
4. Coarse grain (from the perspective of biological organization) phenomena will derive mostly from local component interactions at a finer grain (local includes a living entity's immediate environment). When required, finer grain mechanisms can respond to coarser grain phenomena.
5. Semi-automated modeling methods are needed to more rapidly complete three critical activities:
  - Conduct *in silico* experimentation to explore and shrink spaces of competing mechanistic hypotheses plus alternative mechanism instantiations and parameterizations.
  - Use cross-model validation methods (based on quantitative similarity measures) to discover parsimonious options to increase and decrease component and analog granularity when milestones change and when new questions require new use cases, which necessitates changing targeted attributes and/or shifting attention to new aspects and phenomena.
  - Discover testable hypotheses about how changes in clinical, field, or laboratory measures may be linked mechanistically to observed or observable changes in particular biological level phenomena, especially molecular-level phenomena.

## MECHANISM-BASED APPROACH

New projects are typically initiated based on evidence (even if limited) supporting conceptual mechanistic models (morbidity or disease progression, cause-effect, pharmacology, clinical outcomes, etc.) that often include hypothesized molecular targets. There are large gaps in the mechanistic knowledge landscape that must be filled strategically to facilitate making ‘correct’ go/no-go decisions. The mechanistic landscape spans cell cultures, model organisms, and humans. It also spans pharmacology, toxicology, disposition, metabolism, and more. Today, no one member of the R&D enterprise has a comprehensive ‘view’ of that landscape, yet it is clear from the cited experts that full knowledge of the current state of that landscape and what can be predicted from it is needed at each go/no-go decision juncture. Knowledge of—or use of one or more features on—that mechanism landscape is common to all identified model use cases.

The ability to navigate, use, and leverage current mechanistic insight is a common feature of all paramount use cases. Enabling those use cases requires transitioning from models that are separate and distinct computational (or diagrammatic) descriptions of conceptual representations (e.g., the systems biology, PBPK, and pharmacodynamic models discussed in the cited reviews) to concrete instances of that knowledge. Doing so is feasible using synthetic analogs of mechanisms.^65^ We create analogs by combining (plugging together) specific elements, often varied and diverse, so as to form a coherent biomimetic whole. The analog is synthetic because it is a software mechanism constructed from extant, autonomous components (in this case, executable software components) whose existence and purpose are independent of the model or mechanistic landscape that they comprise. The expectation is that, upon execution, the interacting elements and components—the mechanism undergoing simulation—will exhibit event sequences and outcomes that are measurably similar to counterpart mechanisms within the corresponding wet-lab experiments that will be used to validate the analog. See Yan et al.^119^, Lam and Hunt^113^, Tang and Hunt^116^, Engelberg et al.^118^, and Park et al.^118^, for validated examples of synthetic analogs and their mechanisms, all designed for use center-left on the three lower Figure 1 spectra.

### Exploratory Iteration

One final, critical capability is needed in order to enable the remaining paramount use cases. It must be straightforward to repeat any simulation experiment using a different compound, set of compounds, or no compound. So doing can be accomplished most easily by building analogs using object-oriented software methods and using different mobile objects to map to different compounds of interest. We require that objects representing drugs can be added (or not) and, when ‘inactive’, their presence will not interfere with any already validated mechanism. Examples of that approach are provided in Sheikh-Bahaei and Hunt^71^, Lam and Hunt^113^, Park et al.^118^, Yan et al.^121^, and Sheikh-Bahaei et al.^122^ In those reports, one CE-object maps to a very small amount of referent compound in a small aliquot of measurable material taken from a referent wet-lab experiment, such as culture media, blood, tissue, etc. In time, these CE-objects will carry information that distinguishes one from another, such as structure specifications, particular physicochemical properties, affinities for biological substrates, etc. It follows that any analog component that might feasibly interact with a CE-object must be able to ‘read’ structure specifications and particular physicochemical properties, and use that information to adjust (or not) its interactions with that object.

To enable the latter, analog components will need to be ‘intelligent’ (able to use artificial intelligence methods): they will be programmed with the results of many earlier validation and falsification experiments and can—absent user intervention—arrive at a customized parameterization that determines how they will interact with a new CE-object. To illustrate, imagine that analogs of each of a battery of *in vitro* model systems have achieved degrees of validation for ten compounds. One analog's referent is an *in vitro* system used to characterize metabolic profiles and predict human metabolic clearance. Focus on that analog: upon validation, for each mechanistic event, it retains its parameterizations for all ten CE-objects. The project team needs an answer to this question. Given three, competing, new chemical entities, are their expected metabolic clearance values within a target range? Each analog component, for each unique mechanistic event, can use available framework tools, follow a provided protocol, and construct a predictive map from the space of selected structure specifications and particular physicochemical properties to the space of validated parameterizations. We extend the mapping to the three new compounds and arrive at unique analog parameterizations for each CE-object counterpart. We then conduct analog clearance experiments using each of the three CE-objects. We use the results to predict wet-lab clearance measures. Sheikh-Bahaei and Hunt^71^ described a prototype example of such a protocol. With a knowledge base of only ten compounds, we would have only limited trust in those predictions. However, we would expect to have more trust after doing the same using a knowledge base of fifty compounds. At that stage we could begin characterizing families of compounds as well as particular molecules. As more information is embedded, the knowledge repository increasingly facilitates the work of the domain expert.

The same basic approach can be used to explore expected outcomes of *in silico* toxicology experiments, experiments in analogs of animal disease models, and even analogs of normal and special human cohorts. There would be no technical barriers to simulation experiments that explore possible drug–drug interactions.

### Beyond Paramount Use Cases

Figure 1 helps to bring into focus important challenges faced by any new, high risk, high gain pharmaceutical R&D project. It can be characterized as being left of center on the bottom three spectra. A task is to acquire just enough new information and knowledge (move right in Figure 1) to make better-informed, ‘good’ go/no-go decisions sooner within budget constraints and in the face of considerable uncertainty. The expectation is that simulations will use available knowledge to provide essential information and/or guidance moving forward. To do so, M&S methods must be engaged at the start of the project. Otherwise there will be considerable risk that M&S efforts will lag behind wet-lab efforts. The logical beginning scenario would be to pull together components from models successfully supporting more mature projects, modify them as appropriate, and assemble them to begin being synergistic with wet-lab experiments. Even when the new effort is far left in Figure 1 spectra, speculative and qualitative analogs can be constructed to help researchers think clearly, explicitly, and concretely about the referent and design specific, well-focused wet-lab experiments. That scenario is an example of model use cases beyond those that directly support product development and approval. It also speaks to requirements, covered by those above, which have apparently not yet been considered.

A related, also essential use case will occur once a new product has been approved: revisit the process *in silico* (from start to finish) to explore alternative R&D, knowledge acquisition paths that would have been more time, cost, and/or knowledge effective. To do so will require simulating phases of the R&D process, including various wet-lab experiments performed or not. Such exploratory simulation may seem futuristic, but it is easy to believe that, with such capabilities, the field will have achieved dramatic productivity gains and that is the objective. These additional M&S use cases illustrate that future development of simulation models must take place within a common framework, if the simulations are expected to make a lasting contribution to the knowledge base. Simulated R&D activities will be analogs of past or future, real or considered R&D activities. None of the analogs used, however, will be fully detailed or fully validated. Because we will be center-left on the bottom three Figure 1 spectra, we know that they will (always) be flawed in ways yet to be determined. Those flaws will be hidden by built-in uncertainties.

Simulations that support and add plausible detail to early conceptual models, such as hybrids of systems biology, PBPK, and pharmacodynamic models, will be useful. Simulation models that can also falsify aspects of those conceptual models, earlier rather than later, will be especially valuable because hypothesis falsification, not validation, generates the new knowledge that will be needed for making go/no-go decisions.^123^,^124^ Enabling mechanism falsification in addition to mechanism validation is another, important use case not specifically identified in the cited reviews. Falsification of an analog requires that its mechanism be concrete and particular.^65^ Plausible conceptual mechanisms, especially those that are more complicated, can in theory be falsified using wet-lab experimentation, but doing so can be challenging and time- and resource-intensive.

### Analog Based Knowledge Repository and AB Models

Representing within a knowledge repository the variety of data structures, experimental observations, and documented biological phenomena that may influence go/no-go decisions during the R&D projects is beyond the scope of any one model of computation (MoC). A successful analog based knowledge repository will require the co-existence of multiple, occasionally inconsistent, yet equally valid, models of the same referent(s), as has been done within other domains.^125^ Inconsistency robustness^126^ is important to the aspect-oriented nature of scientific M&S.^65^,^104^,^127^ The management of inconsistent models is part of the strategy for integrating MoCs into a coherent knowledge repository. The choice of underlying MoC for any particular activity is driven by the focal aspects or model (analog or component) use cases. Variety in aspects is better realized by variety in MoCs. For example, a dataflow MoC can co-exist with a discrete event analog of a mouse cancer model, the former representing a functional relationship between high volume population-oriented variables (like concentration in a PBPK model) and the latter representing mechanistic relationships between unique, biomimetic components. For the foreseeable future, such a repository will not, itself, be AB, but it will be partially agent-directed.^115^ Agents such as Experiment Agents used in our work^118^,^128^–^130^ follow protocols and perform tasks exactly analogous to those human modelers perform: set up and execute various models (analogs, modules, etc.) and use pharmacometric methods. Drawing on work from Lee and colleagues^125^,^131^,^132^, we anticipate no restrictions being placed on MoCs, except where required for managed composition, execution, and analysis as has been done with Ptolemy II^125^ for M&S distributed computing scenarios. Depending on use case requirements, analogs may be realized by any given MoC, including continuous-time systems, ABMs, ODEs, systems and process networks, Stream X-Machines, etc.

### Conclusion

An eruption of recent articles, including those cited,^73^–^102^ is focusing attention on the declining productivity crisis stressing pharmaceutical R&D. The hundred plus authors of the more than thirty cited reviews and commentaries argue from different perspectives (pharmacometrics, quantitative and systems pharmacology, model-based drug development, etc.) for expanded use of M&S methods as the primary means to reverse the downward productivity trend. They describe more than ninety particular M&S use cases. The Box 3 includes particular examples. They were merged into paramount use cases listed above. We made the case that facile realization of those use cases will require use of an analog based knowledge repository that fulfills five requirements. AB M&S methods will play important roles along with those methods already in use, but additional capabilities and methods are required. Importantly, there are no technology gaps. The five requirements can be satisfied by the tools listed under Resources, which are in use within other domains.

Reversing the productivity decline requires rethinking how M&S methods can and should be used within the larger R&D process. It requires shifting focus from analysis of data to discovery of explanatory mechanisms. The latter, conditioned paramount use cases, requires implementing the requirements listed above. As illustrated in Figure 1, and as explained in Hunt et al.^104^ and Supporting Information, so doing requires increased reliance on relational grounding and diminished reliance on absolute grounding. It also requires being clear about how computational models are being used and continuously exploring how barriers to participation in M&S activities can be lowered.

**BOX 3**

**CLUSTERED EXAMPLES OF SPECIFIC M&S USE CASES**

These examples (selected randomly) were drawn from those listed in Supporting Information-Use Cases. They illustrate the diversity and scope of particular envisioned^73^–^102^ M&S use cases.

Knowledge integration

Quantitatively address questions concerning the functional relationship between prognostic factors, dosage, and outcomes.^86^,^93^,^96^

Integrate the basic components to describe and understand the complex interplay between the pharmacology of drug action and (patho-) physiological systems.^85^,^93^

Management of uncertainty

Account for uncertainty in the underlying assumptions and thus resulting prediction of drug effects.^76^,^91^

Decision support

Document decision-making from discovery through development to regulatory filing and improve answering postfiling questions and approval, as well as life cycle management of the asset.^96^

Provide a decision-making tool for selecting effective and safe doses, optimizing study sample sizes, evaluating alternative trial designs and making rational go/no-go decisions based on the probability of achieving predefined study goals.^75^,^78^,^79^,^91^,^96^,^97^

Preclinical and clinical development

Facilitate design and/or selection of lead compounds, selection of the first-in-human dose, early clinical trial design, and proof-of-concept studies of experimental drugs and drug combinations.^74^,^75^,^82^,^83^,^85^,^102^

Simulate outcomes of alternative study designs before the experimental investigation commences—incorporating different doses and/or different patients and computing the probability of a successful trial given the characterized patient population and proposed treatment regimens.^75^,^78^,^86^

Predict the toxic potential of chemicals and human adverse effect, and generate hypotheses about the putative molecular mechanisms of chemical-induced injury.^94^,^99^

Drug-disease modeling

Use to understand the relationship between drug effect and the natural progression of the underlying disease.^76^,^85^,^91^,^93^

Describe macrophysiological processes within a particular disease state and use to understand likely modulation of those processes with specific interventions.^73^

*Drug*–*drug interaction, special populations*

Guide investigations in pediatric, the elderly and other special populations.^76^,^84^,^88^–^91^,^95^ Identify and assess complex drug-drug interactions early in drug development so that clinical studies could be planned or prioritized to assess the risk.^79^

Prediction

Allow for extrapolation of the PK properties across species and compounds.^85^,^86^,^89^,^95^

Ultimately predict human PK from *in silico*, *in vitro*, and physicochemical data.^83^

## WEB RESOURCES

Listed below are available, open-source tools of the type that can be used now to begin satisfying the listed interactive Knowledge Repository requirements

**Databases**

PostgreSQL (relational): http://www.postgresql.org/

ZOBD: http://zodb.org/

NoSQL: http://nosql-database.org/

RDF/SPARQL: http://www.w3.org/TR/rdf-sparql-query/

**Data [de]composition**

R: http://www.r-project.org/

HDF: http://www.hdfgroup.org/HDF5/

**Semantics**

Protoge: http://protege.stanford.edu/

Ontopia: http://www.ontopia.net/

Jena: https://jena.apache.org/

Pellet: http://clarkparsia.com/pellet/ (complete)

Racer: http://www.sts.tu-harburg.de/∼r.f.moeller/racer/ (complete)

Hermit: http://hermit-reasoner.com/

**Executable models**

Code

Eclipse: http://eclipse.org/

Netbeans: http://netbeans.org/

Graphical programming

Ptolemy II: http://ptolemy.eecs.berkeley.edu/ptolemyII/index.htm

NetLogo: http://ccl.northwestern.edu/netlogo/

ArgoUML: http://argouml.tigris.org/

Modeling infrastructure

JAMES II: http://wwwmosi.informatik.uni-rostock.de/mosi/projects/cosa/james-ii

CC3D: http://www.compucell3d.org/

MASON: http://cs.gmu.edu/∼eclab/projects/mason/

*Actor model*133

Erlang: http://www.erlang.org/

Scala: http://www.scala-lang.org/node/242

**Workflow**

Kepler: http://kepler-project.org/

Taverna: http://www.taverna.org.uk/

Galaxy: http://galaxyproject.org/

Triana: http://www.trianacode.org/

**Graphs**

NetworkX: http://networkx.lanl.gov/

JUNG: http://jung.sourceforge.net/
