# Supplementary material for: Agent-based modeling: a systematic assessment of use cases and requirements for enhancing pharmaceutical research and development productivity
Source: Wiley Interdiscip Rev Syst Biol Med. 2013 Jun 4;5(4):461–80. doi: 10.1002/wsbm.1222 (PMC3739932; doi:10.1002/wsbm.1222)
Supplement: Supplementary file 1 [file wsbm0005-0461-SD1.doc]

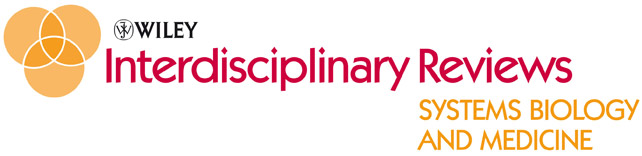
 **Supporting Information**

**Agent-Based Modeling: A Systematic Assessment of Use Cases and Requirements for Enhancing Productivity in Pharmaceutical Research and Development**

Hunt CA, Kennedy RC, Kim SHK, Ropella GEP

**Content** pages

I. Use Cases 1-5

Use Cases References 6-7

II. Supplemental Figure S1 8-9

III. Supplemental Text 10-13

Supplemental Text References 14

**Use Cases**

**Listed below are specific, current, desired, and envisioned modeling and simulation use cases spanning the pharmaceutical R&D enterprise.** The articles referenced are representative of many recent articles that discuss M&S in the context of strategies for reversing the productivity decline.

**Knowledge Integration**

- Integrate knowledge from all aspects of drug development (*in vitro*, *in vivo*, preclinical, clinical and post-marketing), thereby serving as a key decision-making tool, enabling rational, scientifically based choices at critical decision points. [8,9,10,11,12,13,16,18,19,21,22,23,24,28]
- Quantitatively address questions concerning the functional relationship between prognostic factors, dosage, and outcomes. [14,21,24]
  - Integrate/capture causal relationships between disease state, prognostic factors, drug characteristics, individual variability, and drug- and disease-induced changes in biomarkers and clinical end points obtained from all phases of preclinical and clinical development. [1,9,21,12]
  - Integrate sources of variability in exposure-response relationships, which in turn should enable the prospective prediction of the probability of clinical outcomes for specific genotypes and phenotypes and thus eliminate the practice of unguided clinical decisions for pharmacotherapy. [9,20]
- Integrate the basic components to describe and understand the complex interplay between the pharmacology of drug action and the (patho-)physiological systems. [13,21]
  - Allow the integration of the physiological structure, biochemical mechanisms and drug-related properties (e.g., membrane permeability, plasma and tissue protein binding, blood-to-plasma ratio, etc.). [3]
  - Integrate information derived from in vitro systems regarding interaction of a drug with enzymes and/or transporters. [16]
- Effectively leverage existing knowledge and guide future research and experiments in order to optimize drug safety and clinical outcomes. [1,2,4,10,11,12,19]
  - Summarize the knowledge in a ready to apply format. [5]
  - Quantitatively summarize all relevant data at the end of a compound’s full development. [14]
  - Enable artificial intelligence applications to make inferences that would be difficult or impossible to achieve for human scientists. [30]
- Provide computer-based conceptualizations of the mechanism of disease and of the links between drug treatment and observed effects. [6]
  - Provide a comprehensive picture of drug action—both therapeutic and adverse—on cell-, tissue-, and organ-level physiological functions. [9,22]
- Integrate knowledge about growth, development, and maturation of various organs and tissues involved in drug metabolism and elimination across pediatric age groups to predict drug pharmacokinetics in children. [12,11,18]
- Act as a knowledge repository, continuing to be refined and incorporating new data as the candidate molecule progresses into clinical development. [1,2]
  - Provide a framework for integration of data related to drug interaction potential, and their capability to consider the time-varying concentrations of interacting drugs and account for physiological and pharmacokinetic complexities. [2]
  - Incorporate experimental animal data, as well as in-silico-derived and in vitro data, into a coherent framework, from which meaningful and reliable assessments can be made. [8]
  - Provide resources for open-software and open-data, collection and sharing, to facilitate communication and allow for growth of data-driven models [3,24,27,29]
- Integrate knowledge across multiple scales to facilitate the development of drug candidates [9,15,22,23,28]

**Management of Uncertainty**

- Explore both variability and uncertainty in experiments and clinical trial designs. [1]
  - Account for uncertainty in the underlying assumptions and thus the resulting prediction of drug effects. [4,19]
  - Formalize assumptions, and close gaps in data and knowledge. [24]
- Project the uncertainty and the probability of success in development programs. [21]
  - Illustrate and define the uncertainty in demonstrating efficacy and safety with different development programs as assessed by, for example, the probability of achieving the target clinical efficacy outcomes with various study design. [19]
  - Perform a team-based knowledge-gap analysis, which can provide a forecast of the impact of knowledge gaps on the outcome of future clinical trials, thereby providing management with a method to prioritize the laboratory investigations at the beginning of the development program. [6]

**Decision Support**

- Fulfill traditional roles to support labeling and confirm decision. [20]
  - Document decision making from discovery through to regulatory filing and improve answering postfiling questions and approval, as well as life cycle management of the asset. [24]
  - Support regulatory decision-making on chemical safety and risk of toxicity. [27]
- Provide a decision-making tool for selecting effective and safe doses, optimizing study sample sizes, evaluating alternative trial designs and making rational go/no go decisions based on the probability of achieving predefined study goals. [3,6,7,19,24,25]
  - Learn and confirm the key characteristics embedded within a drug candidate’s molecular structure, with the goal of providing explicit, reproducible, and predictive evidence for optimizing drug development plan and resource allocation, enabling critical decision making, and eventually bringing safe and effective medicines to patients. [20,21,30]
- Support decision making on such issues as candidate selection, first-in-human dose finding, assessment of drug-drug interaction potential, and definition of appropriate study designs involving drug-drug interactions or inclusion/exclusion criteria for conducting studies of drugs metabolized by polymorphic enzymes. [7,16]
- Conduct decision analyses of the comparative benefits and risks associated with preventive and treatment strategies. [7,15,24]

**Preclinical and Clinical Development**

- Guide creation of an efficient clinical development strategy. [19]
  - More effectively define potential drug candidates and optimize clinical development by identifying appropriate patients, dose, and optimal clinical trial designs. [1,29]
  - Facilitate design and/or selection of lead compounds, selection of the first-in-human dose, early clinical trial design, and proof-of-concept studies of experimental drugs and drug combinations. [2,3,10,11,13,29]
  - Use to select the studies that would be most relevant to conduct or to replace the ‘guesswork’ that is common when prospective clinical studies are not feasible. [3,16]
  - Help select compounds for clinical trials by integrating all the information for particular compounds into a mechanistic framework where selection can be made based on the expected human PK profile. [11]
- Evaluate/explore the performance of different designs based on the currently available information about the drug before a specific design is selected for the next study. [3,6,11,16,19]
  - Simulate outcomes of alternative study designs before the experimental investigation commences—incorporating different doses and/or different patients and computing the probability of a successful trial given the characterized patient population and proposed treatment regimens. [3,6,14]
  - Predict undesirable effects for safety assessment and support of lead selection and optimization. [27]
  - Help prioritize compounds prior to in vivo experimentation to reduce the number of in vivo animal studies performed in discovery, and ensure appropriate dose selection is made. [11]
  - Predict first-in-human dosage requirements in conjunction with traditional studies in laboratory animals to establish concentration-effect relationships regarding safety. [16]
  - Predict the toxic potential of chemicals and human adverse effect, and generate hypotheses about the putative molecular mechanisms of chemical-induced injury. [22,27]
  - Project drug PK profiles under various scenarios, and help determine whether there is a need for additional studies as part of risk-benefit assessment of new drugs. [11]
  - Evaluate the full time-course of systemic drug and metabolite exposure under all the conditions and situations likely to be encountered during clinical development and beyond. [17]
  - Relate drug exposure, in the compartment measured, to the pharmacological effect at the site of action. [24,26]
  - Use to understand the relationships between absorption and its associated parameters, and the interactions of drug substance properties and bioavailability, to guide the formulation development process. [10]
  - Perform clinical trial simulations to improve the design of future trials. [1,5,11,14,19]
  - Predict long-term safety early in drug development. [5]
- Translate in vitro findings into in vivo efficacy and allow streamlining of dose finding for Phase I and II studies as well as the assessment of new dosing regimens for their likely clinical efficacy and safety. [19]
- Characterize the relationships between the biomarkers and the preclinical/clinical outcomes, placebo effects, a drug’s pharmacologic effects both on and off-target-wise, and trial execution characteristics for both the desired and undesired response. [5,23,25]
  - Guide the identification and development of biomarkers that can serve to predict efficacy and safety during the late stages of preclinical development or the early stages of clinical development. [24]
- Predict the treatment effect as a function of dose, regimen and study design. [19]
  - Evaluate the impact of protocol deviations from a specific design, including the dropout and compliance behavior. [19]
  - Provide guidance for designing better randomized efficacy trials, by determining the appropriate experimental design (dose selection, treatment duration, population selection, etc.) that should maximize separation between side effects and efficacy while still maintaining a clinically meaningful benefit. [14]
  - Assess population variability of absorption after administration of drugs via other routes of extravascular administration (e.g., dermal). [16]
- Predict human testing results by integration of information of physiology and physicochemical properties of an investigational drug. [18]
  - Predict drug clearance, tissue distribution and rate and extend of absorption, and plasma and tissue concentration time profiles. [11,18]
  - Predict exposure/response to a drug and/or toxics not just in the plasma or blood, but also in remote and/or inaccessible compartments such as the brain and tumor tissues. [12,24]
- Provide the starting point for ‘stratified’ and/or personalized medicine. [11,16]
  - Enable binning of individual patient’s drug response into tranches to develop a limited set of appropriate treatment regiments (precision/individualized medicine). [9]

**Drug-Disease Modeling**

- Use to understand the relationship between drug effect and the natural progression of the underlying disease. [4,13,19,21]
  - Explore clinical trial designs and methods of data analysis to differentiate symptomatic versus disease modifying effects. [19]
  - Perform longitudinal analysis of disease progression, with all its components, such as placebo effect, lack of adherence and dropout. [14]
  - Simulate the impact of specific diseases (in individuals, clinical trials, and entire populations), and predict pharmacokinetics in specific disease states defined by etiology and severity. [11,12,17,18]
- Describe macrophysiological processes within a particular disease state and use to understand likely modulation of those processes with specific interventions. [1]
- Incorporate dual or multiple drug effects on disease complementary domains. [14]

**Drug-Drug Interaction, Special Populations**

- Guide investigations in pediatric, the elderly and other special populations. [4,12,16,17,18,19,23]
  - Anticipate pharmacokinetic differences in pediatric patients relative to adult patients and assist in the selection and optimal design of in vivo investigations. [17]
  - Explore “what if” scenarios to determine the most likely cause of altered pharmacokinetics in children. [12]
  - Help design and optimize conductance of clinical trials in special populations such as pediatrics, where optimal planning is needed to minimize the ethical and technical difficulties; for example, in pediatric clinical trials, by suggesting first dose/dose range or optimal sampling times, which is of great importance, as taking blood samples is more difficult than in adults and a main challenge in conducing pediatric clinical trials. [3,12]
  - Use for intra-human scaling from healthy adults to special subpopulations (diseased, the elderly, pediatrics, genetically unique subpopulations) to define differential organ dosimetry as it relates to efficacy and/or safety. [4,11]
- Dynamically evaluate the impact of a DDI with respect to the full concentration-time profiles of the interacting compounds. [17]
  - Assess more complex scenarios involving simultaneous dose-dependent inhibition and induction as well as competition for plasma binding, the inhibitory effects of both parent drug and metabolites. [17]
  - Analyze various elements of complex DDIs involving metabolites and nonlinearity with time. [16]
  - Forecast age-dependency and disease-dependency of pharmacokinetics, explore the variability expected in different patient populations, and provide guidance in the design of DDI studies. [2]
  - Capture the complex interplay between the parent compound and its metabolites involved in time-dependent inhibition (TDI). [2]
- Identify and assess complex DDIs early in drug development so that clinical studies could be planned or prioritized to assess the risk. [7]
  - Detect serendipitous connections between approved drugs and harmful or beneficial interactions when taken by the same patient. [30]

**Prediction**

- Allow for exploration of the PK properties across species and compounds [13,14,17,23]
  - Extrapolate knowledge from one drug class to another and more quickly address new therapeutic targets. [13]
  - Provide interpretation of inter-animal and inter-species PK differences (for instance, owning to different absorption or disposition), and extrapolation to humans. [14]
  - Identify, characterize, and explain the relationships between genetic and epigenetic changes and alterations in targets of interest within species-specific mechanistic contexts. [9]
  - Extend the above characterizations to patients to enable individualized &/or cohort tailored medicine. [9]
- Map formulation components and in vitro release profiles to in vivo PK. [10]
  - Use animal and potentially human ADME data to define the differential dosimetry that may ultimately aid in definition of tolerable daily intake allowances or risk profiles when linked to human exposure. [4]
  - Guide dosing and optimal sampling in first-in-human trials and in human health risk assessment to predict blood and organ concentrations that may be linked to safety end points. [4]
- Quantitatively evaluate temporal influences of covariate combinations (sources of the variability) (including genetics, BSA, age, ethnicity, gender, pregnancy, obesity, environmental factors, smoking, etc.) on mechanisms of interest, including ADME. [16,21,23]
- Predict outcomes of treatment protocols, not just in an “average” individual but in any cohort of interest that. [2,3,16]
  - Describe and/or predict drug PK and target exposure in cohorts characterized by different physiological, pathological, and/or morbidity attributes. [7,11,12,18]
- Ultimately predict human PK from in silico, in vitro and physicochemical data. [11]
  - Predict the drug PK, efficacy and side effects in a given individual with a known genotype make-up for relevant ADME and pharmacology proteins. [11]

**Use Cases References**

Listed in the same order as in the manuscript

1. Allerheiligen SR. Next-generation model-based drug discovery and development: quantitative and systems pharmacology. Clin Pharmacol Ther 88(1):135-137 (2010).
2. Baneyx G, Fukushima Y, Parrott N. Use of physiologically based pharmacokinetic modeling for assessment of drug-drug interactions. Future Med Chem 4(5):681-693 (2012).
3. Bouzom F, Ball K, Perdaems N, Walther B. Physiologically based pharmacokinetic (PBPK) modelling tools: how to fit with our needs? Biopharm Drug Dispos 33(2):55-71 (2012).
4. Edginton AN, Joshi G. Have physiologically-based pharmacokinetic models delivered? Expert Opin Drug Metab Toxicol 7(8):929-34 (2011).
5. Gobburu JV, Lesko LJ. Quantitative disease, drug, and trial models. Annu Rev Pharmacol Toxicol 49:291-301 (2009).
6. Grasela TH, Slusser R. Improving productivity with model-based drug development: an enterprise perspective. Clin Pharmacol Ther 88(2):263-268 (2010).
7. Grillo JA, Zhao P, Bullock J, Booth BP, Lu M, Robie-Suh K, Berglund EG, Pang KS, Rahman A, Zhang L, Lesko LJ, Huang SM. Utility of a physiologically-based pharmacokinetic (PBPK) modeling approach to quantitatively predict a complex drug-drug-disease interaction scenario for rivaroxaban during the drug review process: implications for clinical practice. Biopharm Drug Dispos 33(2):99-110 (2012).
8. Huisinga W, Telgmann R, Wulkow M. The virtual laboratory approach to pharmacokinetics: design principles and concepts. Drug Discov Today 11(17-18):800-805 (2006).
9. Iyengar R, Zhao S, Chung SW, Mager DE, Gallo JM. Merging systems biology with pharmacodynamics. Sci Transl Med 4(126):126ps7 (2012).
10. Jiang W, Kim S, Zhang X, Lionberger RA, Davit BM, Conner DP, Yu LX. The role of predictive biopharmaceutical odeling and simulation in drug development and regulatory evaluation. Int J Pharm 418(2):151-160 (2011).
11. Jones HM, Dickins M, Youdim K, Gosset JR, Attkins NJ, Hay TL, Gurrell IK, Logan YR, Bungay PJ, Jones BC, Gardner IB. Application of PBPK modelling in drug discovery and development at Pfizer. Xenobiotica 42(1):94-106 (2012).
12. Khalil F, Läer S. Physiologically based pharmacokinetic modeling: methodology, applications, and limitations with a focus on its role in pediatric drug development. J Biomed Biotechnol 2011:907461 (2011).
13. Mager DE, Jusko WJ. Development of translational pharmacokinetic-pharmacodynamic models. Clin Pharmacol Ther 83(6):909-912 (2008).
14. Nucci G, Gomeni R, Poggesi I. Model-based approaches to increase efficiency of drug development in schizophrenia: a can’t miss opportunity. Expert Opin Drug Discov 4(8): 837-856 (2009).
15. Prokop A, Michelson S. Development: multiscale CSB—simulation tools. Systems Biology in Biotech & Pharma, SpringerBriefs in Pharmaceutical Science & Drug Development Volume 2: pp 87-102 (2012).
16. Rostami-Hodjegan A. Physiologically based pharmacokinetics joined with in vitro-in vivo extrapolation of ADME: a marriage under the arch of systems pharmacology. Clin Pharmacol Ther 92(1):50-61 (2012).
17. Rowland M, Peck C, Tucker G. Physiologically-based pharmacokinetics in drug development and regulatory science. Annu Rev Pharmacol Toxicol 51:45-73 (2011).
18. Sinha V, Kimko HHC. Recent Developments in Physiologically Based Pharmacokinetic Modeling. Clinical Trial Simulations, AAPS Advances in the Pharmaceutical Sciences Series Volume 1, Part 5: pp 483-499 (2011).
19. Suryawanshi S, Zhang L, Pfister M, Meibohm B. The current role of model-based drug development. Expert Opin Drug Discov 5(4):311-321 (2010).
20. Zhang L, Pfister M, Meibohm B. Concepts and challenges in quantitative pharmacology and model-based drug development. AAPS J 10(4):552-559 (2008).
21. Zhang L, Sinha V, Forgue ST, Callies S, Ni L, Peck R, Allerheiligen SR. Model-based drug development: the road to quantitative pharmacology. J Pharmacokinet Pharmacodyn 33(3):369-393 (2006).
    added from QuantPharmSet4
22. Jack J, Wambaugh J, Shah I. (2013). Systems toxicology from genes to organs. Methods in molecular biology (Clifton, NJ) 930:375-397.
23. Kuepfer L, Lippert J, Eissing T. Multiscale mechanistic modeling in pharmaceutical research and development. Advances in Systems Biology, 543-561 (2012).
24. Wetherington J D, Pfister M, Banfield C, Stone JA, Krishna R, Allerheiligen S, Grasela D. M. Model-based drug development strengths, weaknesses, opportunities, and threats for broad application of pharmacometrics in drug development. J Clin Pharmacol 50(9 suppl):31S-46S (2010).
25. van der Graaf PH, Benson N. Systems pharmacology: bridging systems biology and pharmacokinetics-pharmacodynamics (PKPD) in drug discovery and development. Pharmaceutical research, 28(7):1460-1464 (2011).
26. Morgan P, van der Graaf PH, Arrowsmith J, Feltner DE, Drummond KS, Wegner CD, Street SD. Can the flow of medicines be improved? Fundamental pharmacokinetic and pharmacological principles toward improving Phase II survival. Drug Discov Today. 17(9-10):419-24, 2012.
27. Valerio LG. In silico toxicology for the pharmaceutical sciences. Toxicol Appl Pharm 241(3):356-370, 2009.
28. Vicini P. Multiscale modeling in drug discovery and development: future opportunities and present challenges. Clin Pharm Therap 88(1):126-129, 2010.
29. van der Graaf PH. CPT: Pharmacometrics and Systems Pharmacology. CPT: Pharmacomet Syst Pharmacol 1(9):e8, 2012.
30. Yao L, Evans JA, Rzhetsky A. Novel opportunities for computational biology and sociology in drug discovery. Trends Biotechnol 28(4):161-70 (2010).

**Supplemental Figure S1**


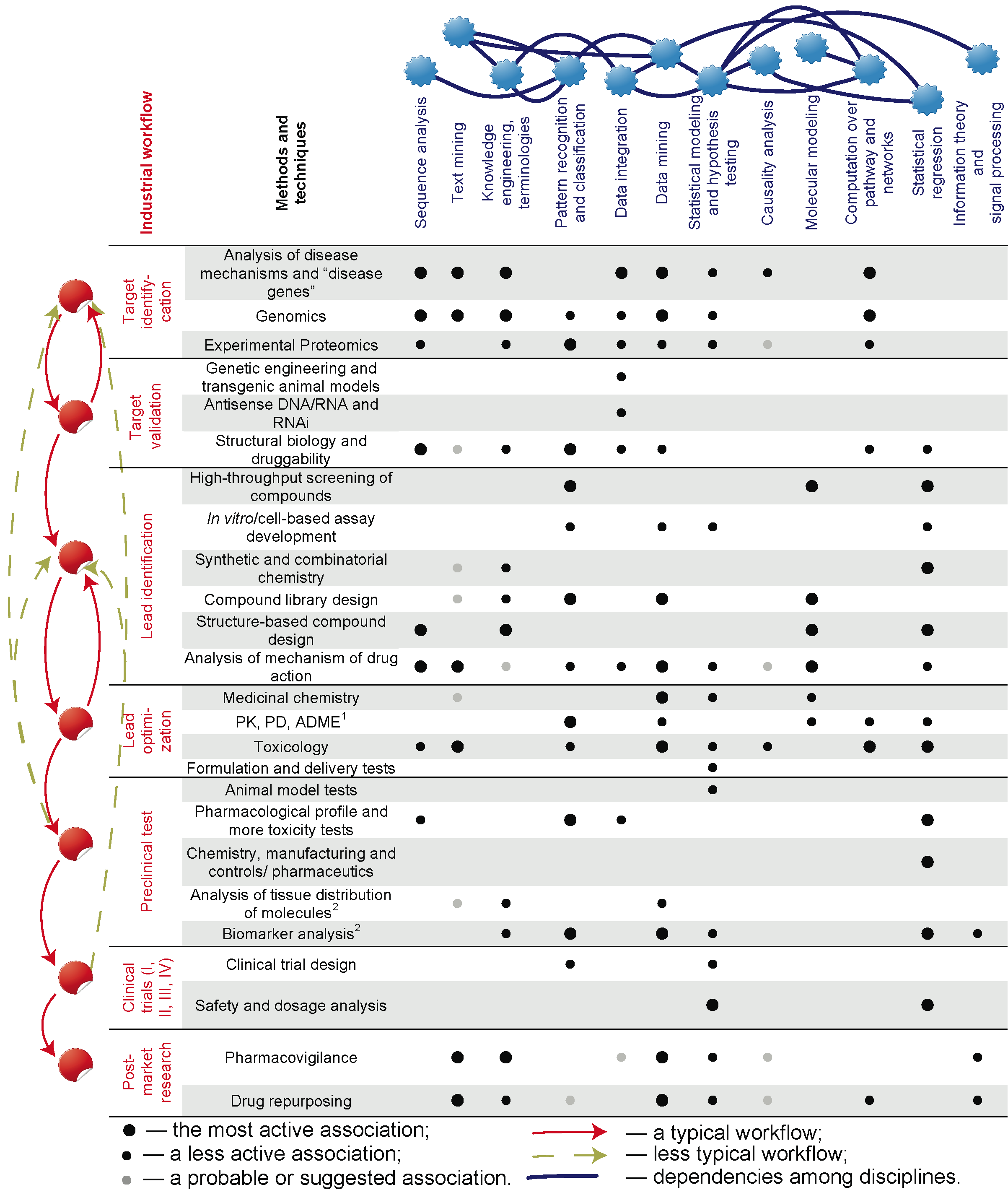


The figure above is reproduced with permission from [Yao L, Evans JA, Rzhetsky A. Novel opportunities for computational biology and sociology in drug discovery. *Trends in Biotechnology* 2010, **28**(4):161-70. doi: 10.1016/j.tibtech.2009.06.003].
The following legend is adapted from the original.

This matrix diagram characterizes various roles of computational technologies within the drug discovery process, and indicates how they impact drug discovery. Traditional linear processes are shifting to become more parallel, simultaneous and cyclical. Red arrows indicate the traditional process and yellow dashed arrows suggest novel workflows that are being used increasingly to increase productivity.

Biomarkers and analysis of the tissue distribution of target molecules are the most recently introduced checkpoints; the FDA does not require them. Computational biology methods are listed along the top. Blue lines illustrate how methods are related.

The impact of each computational technique on each drug discovery stage is classified into three categories: actively or heavily used (large black dot), less actively used (small black dot) and our suggestion (small gray dot).

Chemical informatics is not emphasized because it relates to issues from chemistry and not biology. Chemical informatics comprises a wide range of approaches from computational and combinatorial chemistry that model lead properties and their interaction with targets. Examples include chemical structure and property prediction; structure–activity relationships; molecular similarity and diversity analysis; compound classification and selection; chemical data collection, analysis and management; virtual drug screening; and prediction of in vivo compound characteristics.

**Supplemental Text**

The following text is adapted from Hunt CA, Ropella GEP, Lam TN, Gewitz AD. Relational grounding facilitates development of scientifically useful multiscale models. Theoretical biology & medical modelling 2011B, 8:35. <http://www.tbiomed.com/content/8/1/35/>

**Essential roles of relational grounding in satisfying requirements**

The units, dimensions, and/or objects to which a variable or model constituent refers establish groundings. Absolute grounding: variables, parameters, and I/O are in real-world units. Each term is foundational and maps to a tacit entity having a conceptual or biological meaning. Relational grounding: variables, parameters, and I/O are in units defined by other system components. Variables and components may map to counterparts outside the system or have meanings that are unrelated to the real world. Biology uses relational grounding. Grounding issues do not pose problems for conventional models because they are most often focused narrowly on a single aspect of a system (e.g., a PBPK or gene network model). However, when the model is an analog that aims to describe multiple aspects (i.e., different phenotypic attributes), grounding problems emerge. To satisfy the above requirements, a spectrum of model classes, methods, and groundings will be needed: absolute grounding occupies one extreme; relational grounding occupies the other. The conventional model classes cited in the reviews, mostly pharmacometric and inductive, data-focused ODEs, which use absolute grounding, will remain important and will see critical use cases, but how those methods are used within the framework will differ from those described in the reviews.

Inductive, data-focused, mechanistic, ODE models are accretions of at least three different model types: 1: conceptual models of biological mechanisms, features, and aspects; 2: equations that describe temporal phenomena of type 1; 3: measurement units that provide a quantitative 1:1 mapping from type 2 to measures of biological phenomena. The conceptual models are grounded to the biology via the literature, proprietary information, and expert opinion. Both types 1 and 2 may contain bias and/or questionable assumptions. Good science requires all three be made specific and concrete. Such model accretion reduces flexibility and makes reuse problematic. Progress in satisfying the Box 2 use cases requires a different three-step approach. 1) Map specific biological features to actual, concrete, relationally grounded software objects and spaces that have execution protocols. They will become analogs. 2) Measure actual phenomena generated during executions. 3) Specify quantitative mapping models to relate measures of in silico phenomena to real (not derived: see below) measures of referent phenomena. Such model separation provides the required flexibility and encourages reuse.

Requiring relational grounding is essential because so doing enables synthesizing flexible, easily adapted, extensible, hierarchical analogs of the systems they mimic. Grounding to metric spaces and real world units—absolute grounding—provides simple, interpretive mappings between output, parameter values, and referent data. However, complex issues must be addressed each time one of the following occurs: expand the model to include additional phenomena; combining models to form a larger system; and/or model context changes. Expansions are challenging, even infeasible, when center-left in Fig. 1. A PBPK model, for example will have limited reusability when experimental conditions change or when an assumption made in the original model formulation has been falsified. Reusability is hindered in part because the conflated semi-mechanistic ODE model and the model-to-referent mapping model have different uses. The components and processes in discrete event, object and agent oriented, biomimetic analogs need not have assigned units [1-6].

Grounding decisions impact managing mechanistic and related uncertainties within analogs. In ODE models grounded absolutely, variables and parameters are often expressed as precise mathematical values, even though their networked uncertainties are usually significant. Examples include the physiological parameters in mechanistic PBPK models. Representing uncertainty within such models is mathematically complex and can be problematic for large models. Integrating models from different contexts can require that the whole model be refitted, which should involve re-examination of the cumulative consequences of the networked assumptions. In contrast, probabilistic functions within analogs can represent inherent uncertainties conveniently.

An expectation running through the use cases listed in Supplement—Use Cases is that models will evolve (become more complicated) as R&D advances and new mechanistic insight accumulates, which will require changing, adding, and removing component linkages within analogs. We have recommended [3] striving to keep component groundings relational. So doing facilitates component replacement, limiting any one component formulation solely to its coupling with the others. Any component can be replaced at will as long as the minimal I/O matching requirements are met. Starting with relationally grounded analog components allows the modeler to iterate progressively from qualitative to quantitative validation.

Validation will be based on similarity of phenomena generated during analog simulations to corresponding referent system phenomena. The similarity spectrum can range from qualitative to quantitative. With qualitative similarity, objects will either possess some quality, or they will not. Simulation and referent attributes are considered similar if they have (almost) the same qualities. With quantitative similarity, attributes are categorically the same but vary by magnitude and can be compared by some ordering relation (e.g., less than or greater than). It is always the case that a valid qualitative description is a prerequisite for (and provides constraints for) quantitative descriptions, in the sense that any quantities defined must relate to one or more qualities. Validation (or lack thereof) should act as an important determinant of what type of analog or model (and therefore, what type of grounding) to employ at different stages during project R&D.

Relational grounding facilitates scaling between wet-lab and animal models used during R&D (and between different cohorts of individuals). Consider scaling metabolic clearance of a particular compound in mice (ml/ min/g) to enable human prediction when clearance is grounded absolutely on concentration and time. Scaling to human clearance values requires applying mass, volume, and time scaling factors to all parameters simultaneously, knowing that each scaling factor is imprecise and uncertain. When scaled predictions deviate significantly from observed values (common), there is no way to ascertain which scaling factor(s) and/or which scaled parameter(s) is problematic. Within a relationally grounded analog, mass and volume scaling can be done separately and validated independently. Setting the scale for one variable in accordance with trusted validation data can help set the scale for other, related variables. Time scaling is more complicated [3]. It may require a separate scaling for each probability parameter. However, satisfying the requirements opens the door to using available methods and tools (Box 3) to automate analog-to-analog scaling for analogs grounded relationally.

Relational grounding will also facilitate development of theories of translation. One relationally grounded analog exhibiting the above requirements can be morphed into another. That morphing models conceptual translation and thus stands as a tentative theory of cause-effect translation. It can be directly challenged and falsified (or not). Importantly, gaining exploitable insight into normal-to-disease transitions can be best achieved with analog components that use relational grounding. Park et al. [4] provide an example. Such transitions will require change in how components at multiple levels are parameterized and/or interact. Alternative mechanistic scenarios will need to be explored and challenged. Having components grounded absolutely makes such exploration problematic. Reliance on relational grounding simplifies mechanism exploration and makes the process more intuitive.

Satisfying the requirements requires that all analogs be articulated: consist of distinct parts, modules, and/or components, some of which are autonomous. However, articulation issues are orthogonal to grounding issues. A component that initiates and maintains its own run-time is autonomous. Mammalian cells can be autonomous in vitro. When analog components are quasi-autonomous, they can be effectively replaced by other components for which component I/O requirements are specified in ways consistent with biology. The extent to which a component is autonomous is handled by the clear specification and maintenance of component use cases (aspects, phenotypic attributes, experiment protocols, etc.) or, collectively, by the component’s phenotype. A model will lack any autonomy as long as there is only one use case for given component and a single use case for all connected components. Autonomy can be established regardless of how a module is grounded, but only when targeted phenotypic attributes are clearly defined, which can be challenging when left-of-center in Fig. 1.

Relational grounding facilitates referent knowledge embodiment within analog mechanisms. Computational biology markup languages standardize relationships between the terms. To fulfill the requirements, we must have explicit ontologies enabling specialization into technical programming (Box 3) and biomedical domain expertise. An expert must be able to examine an analog, its components, and simulation events without needing computational expertise. Achieving the knowledge repository will have parallels to the evolution of modern biomedical research. Biomedical scientists design and perform complex experiments without becoming experts in laboratory equipment, reagent design, and production. Progression from custom-built experimental to standardized lab products allowed scientists to effectively compress complicated methods. They were subsumed by engineering, production, and validation processes. That subsumption reduced experimental variability, facilitated experiment replication, and lowered costs, which freed scientists to build increasingly more sophisticated experiments atop complicated equipment (e.g., a cell sorter, a confocal microscope, monoclonal antibodies, transfection reagents, automated DNA sequencer). An analogous progression, progressed in time, must occur for the envisioned productivity gains to be achieved.

**Neither knowledge nor semantics is embedded (encoded) in equation models (e.g., PBPK)**

***Ready to use software tools.*** Bayer’s PK-Sim [7] and its relationship with MoBi [8] provide a good example for understanding where knowledge is encoded in “ready to use” software tools and packages. MoBi is a generic PBPK simulation tool that can express a large variety of specifically designed systems of ODEs based on low-level structures (e.g., molecules and reactions). PK-Sim restricts the set of possible models by providing higher-level structures (e.g., individuals, populations, compounds, etc.). PK-Sim and MoBi are designed so that the more restrictive PK-Sim models can be imported into MoBi and further refined to provide high-level models with use case specific, low-level, modifications.

In order to draw a complete picture of where the knowledge is encoded in such models, we can contrast a PK-Sim model, imported and modified by MoBi against a system of ODEs implemented directly in, say, Matlab. Matlab is an even more generic modeling tool than MoBi. It can not only express PBPK models, but virtually any type of computation. Given these three degrees of expressibility (Matlab, MoBi, and PK-Sim), we see that the higher level constructs act as constraints. The more a set of possible models is constrained to a particular organization of the system of equations and boundary values, the more knowledge is encoded in the system.

Hence, it is the constraining of the set of possible models that encodes the knowledge, not the particular model that obtains at the end of the development process. In other words, it is the PK-Sim and MoBi tools that encode the knowledge, not the equations they numerically integrate during simulation.

***Where is the knowledge?*** The biomedical domain knowledge that one associates with a PBPK model is found in the interpretations of equations, provided by domain experts, found in the domain literature. Those interpretations provide a semantic grounding of otherwise generic equation models. The semantic grounding of such equations is always separate from their software implementations. However, given “ready to use” software tools of the type described above, it is easy for a “user” of those tools to conflate the knowledge with the equations. It is a good practice to keep them separate. The framework described in the main text will facilitate doing so.

To illustrate the above point, consider Garg and Balthasar’s PBPK model of immunoglobulin-gamma (IgG) kinetics [9]. The authors aimed to predict the distribution of an antibody. The model was discussed in example one in [10]. The authors clearly describe their model in prose supported by sketches. Straightforward ODEs with parameterization details are provided. Variables within their model take meaning regardless of the background of the reader. For example, Equation 2 from [9], shown below, describes the rate of change of IgG plasma concentration. A software engineer (SE) with no biological knowledge would be able to draw on her knowledge of mathematics to build an executable implementation of the model. It is probable that she would first rewrite the equation shown below in pseudocode to facilitate translation to a programming language. The variables in the equation have no immediate meaning to the SE. Her code can be implemented absent any biomedical knowledge. A domain expert typically supplies parameterizations and draws on domain knowledge to interpret the input-output (I/O) using prose and sketches thus providing the semantic grounding. Within “ready to use” tools, separate software features can mimic the domain expert’s activity and provide standardized interpretations. It thus becomes clear that neither knowledge nor semantics is embedded in those equations.

Let Lu = Lung, P = Plasma, Lv = Liver, S = Spleen, H = Heart, K = Kidney, Sk = Skin, M = Muscle,

O = Others

V_P_*C_P_dt = (Q_Lu_-L_Lu_)*CV_Lu_ – ((L_GI_+L_S_+Q_LV_+Q_H_+Q_K_+Q_Sk_+Q_M_+Q_O_)*C_P_) + ((1-sigma_Lu_)*L_Lu_*CI_Lu_) + ((1-sigma_GI_)*L_GI_*CI_GI_) +
 ((1-sigma_S_)*L_S_*CI_S_) + ((1-sigma_Lv_)*L_Lv_*CI_Lv_) + ((1-sigma_H_)*L_H_*CI_H_) + ((1-sigma_K_)*L_K_*CI_K_) +
 ((1-sigma_Sk_)*L_Sk_*CI_Sk_) + ((1-sigma_M_)*L_M_*CI_M_) + ((1-sigma_O_)*L_O_*CI_O_)

**References**

[1-7] and [10] are cited in the main text.

1. Engelberg JA, Datta A, Mostov KE, Hunt CA. MDCK cystogenesis driven by cell stabilization within computational analogues. PLoS Computational Biology 2011, 7(4):e1002030. doi: 10.1371/journal.pcbi.1002030.
2. Hunt CA, Ropella GE, Lam TN, Tang J, Kim SH, Engelberg JA, Sheikh-Bahaei S. At the biological modeling and simulation frontier. Pharmaceutical Research 2009, 26:2369-2400. doi: 10.1007/s11095-009-9958-3.
3. Hunt CA, Ropella GEP, Lam TN, Gewitz AD. Relational grounding facilitates development of scientifically useful multiscale models. Theoretical Biology and Medical Modelling 2011, 8:35. doi: 10.1186/1742-4682-8-35.
4. Park S, Kim SH, Ropella GEP, Roberts MS, Hunt CA. Tracing multiscale mechanisms of drug disposition in normal and diseased livers. Journal of Pharmacology and Experimental Therapeutics 2010, 334(1):124-136. doi: 10.1124/jpet.110.168526.
5. Sheikh-Bahaei S, Maher JJ, Hunt CA. Computational experiments reveal plausible mechanisms for changing patterns of hepatic zonation of xenobiotic clearance and hepatotoxicity. Journal of theoretical biology 2010, 265:718-733.
6. Tang J, Hunt CA. Identifying the rules of engagement enabling leukocyte rolling, activation, and adhesion. PLoS compututational biology 2010, 6(2):e1000681. doi: 10.1371/journal.pcbi.1000681.
7. <http://www.systems-biology.com/products/pk-sim.html>
8. <http://www.systems-biology.com/products/mobi.html>
9. Garg A, Balthasar JP. Physiologically-based pharmacokinetic (PBPK) model to predict IgG tissue kinetics in wild-type and FcRn-knockout mice. Journal of Pharmacokinetics and Pharmacodynamics 2007, 34:687-709. doi: 10.1007/s10928-007-9065-1.
10. Hunt CA, Ropella GEP, Lam TN, Gewitz AD. Relational grounding facilitates development of scientifically useful multiscale models. Theoretical Biology and Medical Modelling 2011, 8:35. doi: 10.1186/1742-4682-8-35.
